# Molecular screening of patients with nonsyndromic hearing loss from Nanjing city of China^☆^

**DOI:** 10.1016/S1674-8301(11)60042-0

**Published:** 2011-09

**Authors:** Yajie Lu, Dachun Dai, Zhibin Chen, Xin Cao, Xingkuan Bu, Qinjun Wei, Guangqian Xing

**Affiliations:** a Department of Biotechnology, Nanjing Medical University, Nanjing, Jiangsu 210029, China;; b Department of Otolaryngology, the First Affiliated Hospital of Nanjing Medical University, Nanjing, Jiangsu 210029,China.

**Keywords:** nonsyndromic hearing loss, *GJB2*, *GJB3*, *GJB6*, *SLC26A4*, *SLC26A5*, mitochondrial DNA, gene mutation

## Abstract

Hearing loss is the most frequent sensory disorder involving a multitude of factors, and at least 50% of cases are due to genetic etiology. To further characterize the molecular etiology of hearing loss in the Chinese population, we recruited a total of 135 unrelated patients with nonsyndromic sensorineural hearing loss (NSHL) for mutational screening of *GJB2, GJB3, GJB6, SLC26A4, SLC26A5* IVS2-2A>G and mitochondrial *12SrRNA, tRNA^Ser(UCN)^* by PCR amplification and direct DNA sequencing. The carrier frequencies of deafness-causing mutations in these patients were 35.55% in *GJB2*, 3.70% in *GJB6*, 15.56% in *SLC26A4* and 8.14% in mitochondrial *12SrRNA*, respectively. The results indicate the necessity of genetic screening for mutations of these causative genes in Chinese population with nonsyndromic hearing loss.

## INTRODUCTION

Hearing loss is the most common sensory disorder in humans. Profound hearing loss affects approximately one in 1,000 live births in the general population, and 50%-60% of these cases have genetic etiologies[1]. Genetic hearing loss can follow a pattern of autosomal dominant, autosomal or X-linked recessive, or mitochondrial inheritance. About 70% are classified as nonsyndromic since hearing loss is the only symptom, while 30% are classified as syndromic and are associated with other clinical features[2].

There are more than 150 genetic loci that have been described for nonsyndromic sensorineural hearing loss (NSHL) in humans, and about 60 of them were cloned (Hereditary Hearing Loss Homepage: http://hereditaryhearingloss.org). It is believed that alterations in several members of the connexin protein family, mutations in the solute carrier 26 (SLC26) family and in the mitochondrial DNA (mtDNA) contribute to the development of the majority of genetic hearing losses[2]–[8].

It is estimated that there are approximately 20 million babies born every year in China, of whom about 30,000 are expected to have congenital hearing loss[9]. Carrier frequencies of some mutational hot spots associated with NSHL such as *GJB2* 235delC and mtDNA A1555G have been reported, but the molecular etiology of NSHL in Chinese population has not been investigated completely in most areas. In this study, we screened the *GJB2, GJB3, GJB6, SLC26A4, SLC26A5* IVS2-2A>G and mitochondrial genes to determine the etiology of hearing loss in eastern China.

## MATERIALS AND METHODS

### Subjects

All the individuals were recruited to participate in the study by signing a written informed consent, and the study protocol was approved by the Ethical Committee of the Nanjing Medical University for Human Studies, and the participants were asked to donate a blood sample. The genetic studies were conducted on two groups: a case group with moderate to profound and nonsyndromic sensorineural hearing impairment (*n* = 135) aged 7-12 (11.3±2.6) years, and a control group (*n* = 162) with normal hearing aged 8-14 (11.5±2.9) years. The female-male ratio of these groups are 74/61 and 75/87, respectively. One hundred thirty-five blood samples were obtained from a panel of sporadic hearing-impaired individuals from Nanjing City School for Deaf Children, and 162 control blood samples were gotten from a panel of unaffected individuals in Jiangsu province. All subjects were Han Chinese in origin, and were evaluated through otological examination and audiological evaluations including pure-tone audiometry (Madsen Orbiter 922), immittance (Madsen Zodiac 901), auditory brainstem response (ABR, Interacoustic EP25), and transient evoked otoacoustic emissions (Madsen Celesta 503). Hearing impairment was defined by the level of hearing loss in the better ear for pure-tone threshold average in the speech frequencies 0.5, 1, 2, and 4k Hz. Hearing loss of 26-40 dB was considered mild; 41-60 dB, moderate; 61-80 dB, severe, and more than 80 dB, profound.

### Molecular screening

The mutations on the *GJB2, GJB3, GJB6, SLC26A4, SLC26A5* IVS2-2A>G and mitochondrial genes were selected for molecular screening. Genomic DNA was isolated from 2 mL of peripheral leukocytes of all participants using Puregene DNA Isolation Kits (DNA fast 2000, Shanghai China). The DNA fragments spanning the entire coding region of those genes were PCR amplified by using reference primers[8],[9]. The quality and quantity of purified genomic DNA were determined by running a 0.8% agarose gel and spectrophotometry. Each fragment was analyzed by direct sequencing in an Applied Biosystems 3730 automated DNA sequencer. The resultant sequence data were compared with the wildtype *GJB2* (GenBank Accession No. GI62999485), *GJB3* (GenBank Accession No.NM_024009), *GJB6* (GenBank Accession No. NT_009799.12), *SLC26A4* (GenBank Accession No. NM_000441.1), *SLC26A5* (GenBank Accession No. AC 005064), and mtDNA (GenBank accession No. NC_001807.4) gene sequences to identify the mutations.

### Data compilation

To determine the most common gene mutations in China, many data in several typical areas of China were reviewed. To examine the possible role of the nuclear genes in patients with non-syndromic hearing loss, we compared the carrier frequencies of *GJB2, GJB3, GJB6* and *SLC26* family mutations, which are common deafness-associated nucleotide changes, from different areas of the world.

## RESULTS

### Molecular analysis

All the results were compared with the standard sequences of *GJB2, GJB3, GJB6, SLC26A4, SLC26A5* and mtDNA *12SrRNA, tRNA^Ser(UCN)^*. A series of sequence variations were detected in these genes as shown in ***Table 1*** and ***Table 2***.

**Fig. 1:**
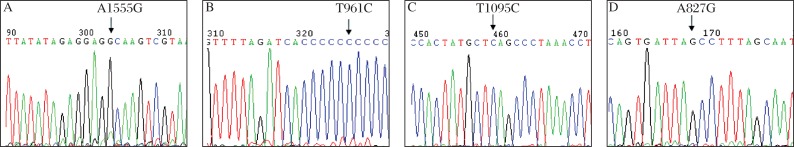
Partial sequence chromatograms of mtDNA *12SrRNA* from the patients. Arrows indicate the location of the base changes. A: A to G transition at position 1555. B: T to C transition at position 961. C: T to C transition at position 1095. D: A to G transition at position 827.

In the case group, there were totally eleven mitochondrial *12SrRNA* sequence variations detected. Of those, four variants are known deafness-associated mutations (***Fig. 1***) with variable frequencies of 1.48% in A1555G, 4.44% in A827G, 1.48% in T961C and 0.74% in T1095C (cosegregate with A1555G mutation). All of these mutations were not found in the control group. One variant, G709A, which was reported to be a polymorphism in some reports, was detected both in the case group (2.22%) and the control group (1.23%). Other variants of mtDNA 12SrRNA, such as T1005C, C1048T, T1119C, C752T, A1382G and A1438G, seem to be polymorphisms rather than causes of disease. On the other hand, we did not find C1494T mutation in the 12SrRNA and any of the known deafness-associated mutations in tRNA^Ser(UCN)^, such as A7445G, 7472insC, T7510C, T7511C, T7512C and G7444A, in all individuals.

**Fig. 2:**
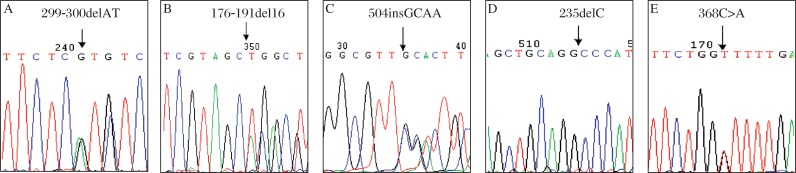
Partial sequence chromatograms of *GJB2* from the patients. Arrows indicate the location of base changes. A: AT deletion at position 299-300. B: 16-base deletion at position 176-191. C: GCAA insert at 504. D: C deletion at position 235. E: C to A transversion at position 368.

Compared to the standard sequence of *GJB2*, we identified twelve types of sequence changes in 88 patients, of which 5 types of variations (299-300delAT, 176-191del16, 504insGCAA, 235delC, and 368C>A) (***Fig. 2***) in 48 patients were thought to be pathogenic, and 4 belonged to benign variant (79G>A, 101T>C, 341A>G and 608TC>AA). The carrier frequencies of deafness-causing *GJB2* mutations in our case group were 0.74% for 176-191del16, 27.41% for 235delC, 4.44% for 299-300delAT, 1.48% for T123N and 1.48% for 504insGCAA, respectively. In addition, the pathogenicity of the other 3 kinds of variants (79G>A+341A>G, 79G>A+109G>A, and 109G>A) have not come to an agreement. Thus, the total carrier frequency of deafness-causing *GJB2* mutations in our case group was 35.55% (48/135) at least. The 235delC appeared to be the most common deafness-related *GJB2* mutation (37/135, 27.41%) with the highest allele frequency of 20.37% (***Table 2*** and ***Table 3***). In the control group, only five subjects were found to carry the heterozygous deafness-causing mutation.

**Table 1:** Variations in mtDNA identified in the subjects

Genetype	Nucleotide change	Amino acid change	Mutation type	Category	Patients (*n*)		Controls (*n*)	
					Homo-plasmy	Heteio-plasmy	Homo-plasmy	Heteio-plasmy
Mitochondrial	709G>A	*12SrRNA*	Missense	undefined	3	0	2	0
	752C>T	*12SrRNA*	Missense	polymorphism	4	0	1	0
	827A>G	*12SrRNA*	Missense	pathogenic	6	0	0	0
	961T>C	*12SrRNA*	Missense	pathogenic	2	0	0	0
	1005T>C	*12SrRNA*	Missense	polymorphism	6	0	3	0
	1048C>T	*12SrRNA*	Missense	polymorphism	2	0	0	0
	1095T>C	*12SrRNA*	Missense	pathogenic	1	0	0	0
	1119T>C	*12SrRNA*	Missense	polymorphism	3	0	0	0
	1382A>G	*12SrRNA*	Missense	polymorphism	4	0	3	0
	1438A>G	*12SrRNA*	Missense	polymorphism	125	0	112	0
	1555A>G	*12SrRNA*	Missense	pathogenic	2	0	0	0
	7445A>G	*tRNA*^Ser(UCN)^	Missense	pathogenic	0	0	0	0
	7472insC	*tRNA*^Ser(UCN)^	Missense	pathogenic	0	0	0	0
	7511T>C	*tRNA*^Ser(UCN)^	Missense	pathogenic	0	0	0	0
Subtotal					158	0	121	0
DM subtotal					11		0	

DM: definite deafening mutations.

**Table 2:** Variations in *GJB2, GJB3, GJB6, SLC26A4* and *SLC26A5* genes identified in the subjects

Gene	Nucleotide change	Amino acid change	Domain	Mutation type	Category	Patients (*n*)			Controls (*n*)		
						Homo	Hetero	Allele frequency(%)	Homo	Hetero	Allele frequency(%)
*GJB2*	79G>A	V27I	TM1	Missense	Polymorphism	4	10	6.67	13	20	17.04
	79G>A+341A>G	V27I+E114G	TM1+IC2	Missense	Undefined	3	2	2.96	1	1	1.11
	79G>A+109G>A	V27I+V37I	TM1	Missense	Undefined	0	1	0.37	0	0	0.00
	109G>A	V37I	TM1	Missense	Undefined	2	5	3.33	0	3	1.11
	101T>C	M34T	TM1	Missense	Polymorphism	0	1	0.37	0	0	0.00
	176-191del16	176-191del16	EC1	Deletion	Pathogenic	0	1	0.37	0	1	0.37
	235delC	235delC	TM2	Deletion	Pathogenic	18	19	20.37	0	2	0.74
	299-300delAT	299-300delAT	IC2	Deletion	Pathogenic	0	6	2.22	0	1	0.37
	341A>G	E114G	IC2	Missense	Polymorphism	3	7	4.81	1	5	2.59
	368C>A	T123N	IC2	Missense	Pathogenic	1	1	1.11	0	1	0.37
	504insAAGG	504insAAGG	EC2	Insertion	Pathogenic	0	2	0.74	0	0	0.00
	608TC>AA	I203K	TM4	Missense	Polymorphism	0	2	0.74	0	8	2.96
*GJB3*	357C>T	N119N	IC2	Samesense	Polymorphism	0	2	0.74	0	0	0.00
	866G>A		3′UTR	Missense	Polymorphism	0	6	2.22	0	1	0.37
*GJB6*	232 kb del	Frameshift	TM3	Deletion	Pathogenic	4	1	3.33	0	0	0.00
*SLC26A4*	IVS7-2A>G	Splice site	intravening	Missense	Pathogenic	7	11	9.26	0	0	0.00
	IVS7-2A>G+	Splice site + H723R	sequence7	Missense	Pathogenic	0	2	0.74	0	0	0.00
	2168A>G		intravening								
	2167 C>G	H723D	sequence7 STAS	Missense	Pathogenic	0	1	0.37	0	0	0.00
*SLC26A5*	IVS2-2A>G	Splice site	intravening sequence2	Missense	Pathogenic	0	0	0.00	0	0	0.00

UTR: untranslated region; IC: intracellular; TM: transmembrane; EC: extracellular; STAS: sulfate transporter and anti-sigma antagonist

**Table 3:** Classification of variants in *GJB2, GJB3, GJB6, SLC26A4* and *SLC26A5* genes identified in all subjects

Variant classification		Patients (*n*= 135)			Controls (*n* =162)		
		Homo	Hetero	Total	Homo	Hetero	Total
AR	*GJB2*	19	29	48	0	5	5
	*GJB3*	0	0	0	0	0	0
	*GJB6*	4	1	5	0	0	0
	*SLC26A4*	7	14	21	0	0	0
	*SLC26A5*	0	0	0	0	0	0
Polymorphism and AR and undefined	*GJB2*	31	57	88	15	42	57
	*GJB3*	0	8	8	0	1	1
	*GJB6*	4	1	5	0	0	0
	*SLC26A4*	0	0	0	0	0	0
	*SLC26A5*	0	0	0	0	0	0

AR: autosomal recessive.

**Fig. 3:**
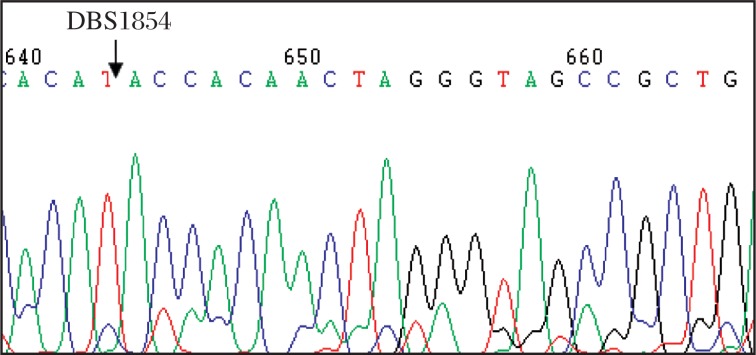
Partial sequence chromatograms of *GJB6* from the patient. The arrow indicates the location of 232 kb deletion (GJB6-D13S1854 mutation).

Apart from *GJB2* mutations, one kind of deafness-causing *GJB6* mutation (*⊿GJB6*-D13S1854 or 232 kb del, ***Fig. 3***) was identified in 5 of 135 hearing-impaired individuals. The heterozygous gene variation frequency of *GJB6* and *GJB2* was 5/135 (3.70%), *GJB2* and mtDNA was 3/135 (2.22%). All of the patients with*⊿GJB6*-D13S1854 were also found to have the 235delC mutation in *GJB2*. None of the normal-hearing person in the control group carries the *GJB6* mutation.

**Fig. 4:**
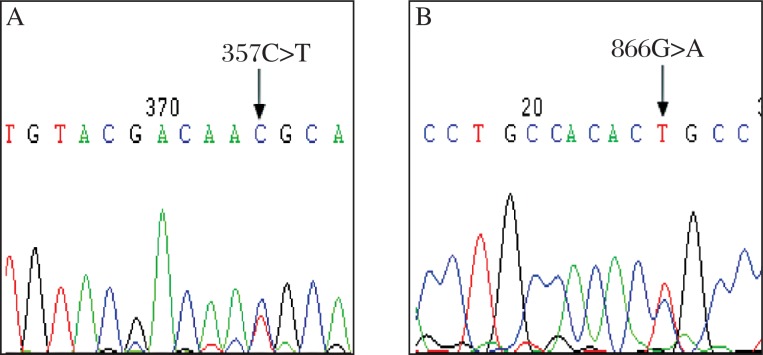
Partial sequence chromatograms of *GJB3* from the patients. Arrows indicate the location of base changes. A: C to T transition at position 357. B: G to A transition at position 866.

In this observation, none of the deafness-causing *GJB3* mutation was detected in both the case and control groups by sequence analysis. Alternatively, we identified two polymorphic variants of the *GJB3* gene, 866G>A (2.22%) and 357C>T (0.74%), as shown in ***Fig. 4*** and ***Table 2***, which were, however, absent in the 162 matched normal-hearing controls.

**Fig. 5:**
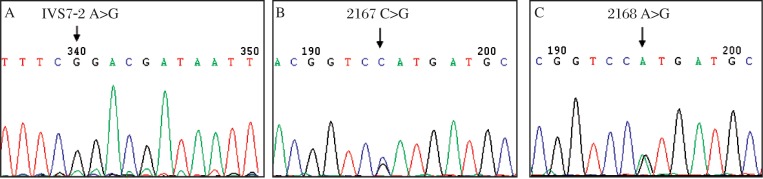
Partial sequence chromatograms of *SLC26A4* from the patients. Arrows indicate the location of base changes. A: A to G transition at position IVS7-2. B: C to G transversion at position 2167. C: A to G transition at position 2168.

There were 3 different kinds of *SLC26A4* mutations (***Fig. 5***) with variable frequencies of 13.33% in IVS7-2A>G, 1.48% in IVS7-2A>G+2168A>G, and 0.74% in 2167C>G, which was reported for the first time. The total carrier frequency of deafness causing *SLC26A4* mutations was 15.55% (21/135) in the case group and no *SLC26A4* mutation was found in the control group. The SLC26A5 IVS2-2A>G variant, however, was not found in a total of 297 individuals with either impaired or normal hearing by sequence analysis.

### Data congregate

Based on the widely and systematically collected information about NSHL from several typical areas of China, we summarize the existing reports on molecular epidemiology of deafness. The prevalence of the *GJB2, GJB3, GJB6, SLC26A4* and mtDNA genes in Chinese populations is listed in ***Table 4***. As for genetic factors of nonsyndromic deafness in China, the average value of variant in *GJB2* seems to be the most common (23.37%), followed by *SLC26A4* (14.74%), mtDNA *12SrRNA* A1555G ( 2.44%), *GJB3* (1.97%) and *GJB6* (1.33%) genes.

After the systematic comparison of various genes in different countries or ethnic differences between the mutation frequencies, we calculated the average frequency of *GJB2, GJB3, GJB6, SLC26A4* and mtDNA mutations in patients with nonsyndromic hearing loss. As shown in ***Table 5***, for hearing-impaired people in the world, the *GJB2* mutation is also the most common causative factor. The variant accounts for about 29.03%, followed by the *SLC26A4* (15.16%) and *GJB6* (5.58%) mutations. The *GJB3* mutation and mtDNA *12SrRNA* A1555G, however, seem not to play an important part in the causative effects of deafness in Western countries as compared with the Chinese people.

## DISCUSSION

*GJB2* (OMIM No.121011) encodes the gap junction protein connexin 26 (CX26), which is expressed in the cochlea and may play a role in K^+^ circulation between different partitions in the cochlea. Both in China and many other countries, *GJB2* mutations are responsible for a large proportion of NSHL. For example, the 35delG, 167delT, 235delC and R143W alleles are the most common *GJB2* mutations in Europeans, Americans, Africans and Asians[10]–[17]. In the Asian nonsyndromic hearing-impaired populations, the 235delC of *GJB2* are 34.0% and 14.3% in two Japanese reports[3],[10], 8.52% and 6.70% in two studies in Taiwan[11],[12], 5.10% in one Korean study[13], and 27.41%(37/135) in our study. In our observation on patients with NSHL from eastern China, 235delC mutation of *GJB2* was higher than that in the Caucasians. None of 35delG was detected in both the case and control groups of our country; however, the 35delG of *GJB2* occupied the main cause of deafness in the Caucasian populations. Therefore, in different ethnic groups, there was a significant difference in genetic mutation between Mongolians and Caucasians.

The Chinese population is made up of several major ethnicities, such as Han, Man, Mon, Hui, Zhuang and Miao. All of the subjects included in our study were Han Chinese in origin, which is the predominant ethnicity (85.56%) of Chinese. Previous studies demonstrated that there was no significant difference of *GJB2* mutations between Han and other minorities through the comparative analysis of reports from six Chinese typical areas (***Table 4***).

**Table 4:** Prevalence of *GJB2, GJB3, GJB6, and SLC26A4* genes and mtDNA *12SrRNA* A1555G mutation in the Chinese population

Different areas of China	Patients (*n*)	Frequency of pathologic variants(%)					References
		*12SrRNA* A1555G	*GJB2*	*GJB3*	*GJB6*	*SLC26A4*	
Northern China	743	2.92	18.12	-	-	18.15	Yu Fei *et al*.2006[25]
							Liu Xin *et al*.2006[26]
							Song RD *et al*.2007[27]
							Zhu QW *et al*.2007[28]
							Zhu YH *et al*.2008[29]
Northeastern China	377	1.00	20.80	-	-	-	Yu Fei *et al*.2006[25]
							Liu Xin *et al*.2006[26]
							Chen JX *et al*.2007[30]
							Wang Ping *et al*.2001[31]
Eastern China	135	1.50	35.55	0	3.70	15.56	This study
Southern China	404	2.70	10.80	-	-	-	Yu Fei *et al*.2006[25]
							Liu Xin *et al*.2006[26]
Northwestern China	1639	4.10	17.61	2.20	0	12.85	Dai Pu *et al*.2006[32]
							Guan Jing *et al*.2006[33]
							Yu Fei *et al*.2006[25]
							Liu Xin *et al*.2006[26]
							Yuan YY *et al*.2008[34]
							Guo YF *et al*.2008[35]
							Du RL *et al*.2009[36]
Southeastern China	140	-	40.00	-	-	-	Wang SH *et al*.2009[37]
Others	533	-	20.70	3.70	0.30	12.38	Li QZ *et al*.2005[38]
							Han DY *et al*.2006[39]
							Li Qi *et al*.2007[40]
							Yuan YY *et al*.2007[41]
							Sun Qing *et al*.2008[18]
Average value		2.44	23.37	1.97	1.33	14.74	43.57% (total)

**Table 5:** Prevalence of *GJB2, GJB3, GJB6, and SLC26A4* genes and mtDNA *12SrRNA* A1555G mutation in different countries

Different areas	Samples(*n*)	Frequency of pathogenic mutations					References
		*12SrRNA* A1555G	*GJB2*	*GJB3*	*GJB6*	*SLC26A4*	
Argentina	252	0.00	41.27	-	1.59	-	Viviana Dalamón *et al*. 2010[42]
Austria	122	0.00	65.57	0.00	16.39	-	RENÉ UTRERA *et al*. 2007[43]; Reinhard Ramsebner *et al*. 2007[20]
Brazil	300	-	13.67	0.00	1.00	-	Ana Carla Batissoco *et al*. 2009[44]
Croatia	121	-	38.84	-	0.00	-	Ivona Sansovic *et al*. 2009[14]; Igor Medica *et al*. 2005[45]
Poland	1313	-	17.75	-	-	-	Agnieszka Pollak *et al*. 2007[46]
Germany	164	0.60	1.22	-	-	-	Li R *et al*. 2004[47]
Greek	30	-	33.33	-	0.00	-	Vassos Neocleous *et al*. 2006[24]
India	484	-	6.82	-	-	5.40	Park HJ *et al*. 2003[7]; Padma G *et al*. 2009[48]
Korea	176	-	37.50	-	0.00	40.00	KY Lee *et al*. 2008[13]; Park HJ *et al*. 2003[7]
Morocco	116	-	24.14	0.00	-	-	RENÉ UTRERA *et al*. 2007[43]
Hungary	410	-	54.88	0.24	0.49	-	TÍMEA TÓTH *et al*. 2007[19]
Spain	152	-	23.40	-	27.66	27.00	Del Castillo F J *et al*. 2010[22]; Pera A *et al*. 2008[49]
Turkey	418	-	24.80	-	0.00	1.70	Sirmaci A *et al*. 2006[23]; Duman D *et al*. 2010[5]
United Kingdom	160	-	22.22	-	22.22	3.50	Del Castillo F J *et al*. 2005[22]; Hutchin T *et al*. 2008[16]
USA	464	0.00	28.44	-	0.00	12.55	Joy Samanich *et al*. 2007[50]; Dai Pu *et al*. 2009[9]
Venezuela	40	-	27.50	-	2.50	-	RENÉ UTRERA *et al*. 2007[43]
Average value		0.15	28.83	0.06	5.99	15.03	50.06% (total)

*GJB3* (OMIM No. 603324) encodes the gap junction protein connexin 31(Cx31), which is also thought to be a good candidate for hereditary hearing impairment. Mutations of *GJB3* cause three different disorders: nonsyndromic deafness, syndromic deafness, and a genodermatosis. More than 10 mutations in *GJB3* have been found in patients with deafness from China[18], Hungary[19], and Brazil[6]. In contrast, several studies demonstrated that the variations in GJB3 with no or low genetic relevance in Moroco and in Austrialia[15],[20]. In our study, two kinds of variants in *GJB3* were detected from 135 hearing-impaired subjects: the 866G>A in three and the 357C>T in one patient. Both of the *GJB3* variations were heterozygotes, which had previously been reported as polymorphisms. However, we did not find these changes in the 162 matched normal-hearing controls. This led us to the assumption that these variants may be associated with autosomal-dominantly nonsyndromic hearing loss in some patients.

*GJB6* (OMIM No. 604418) encoding connexin 30 (Cx30) was an obvious candidate gene for deafness owing to its chromosomal location at 13q12, and because connexin 26 and connexin 30 are expressed in the same inner ear structures and share 77% homology in amino acid sequence[21]. Despite the high prevalence of *GJB2* and *GJB6* mutations in some Western populations, for example, two large deletions of the *GJB6* (one of 309 kb,*⊿GJB6*-D13S1830 and another of 232 kb,*⊿GJB6*-D13S1854) upstream the *GJB2* are frequently found among individuals who are deaf in Spain[22], these mutations seem to account for a smaller percentage of hereditary hearing loss in Turkey[23], Greek Cyprus[24] and Austria[20], and few data have been reported on the presence of these mutations in the Chinese population[8]. In the present study, we assessed the prevalence of *GJB6* mutations in both hearing-impaired individuals and normal-hearing controls from Nanjing city. In five of the 135 patients (3.70%), the 232 kb del (*⊿GJB6*-D13S1854) was detected, one was heterozygote and others were homozygotes. All of them were also found to have the 235delC mutation in *GJB2*. None of the patients negative for *GJB2* mutations carried this mutation. The result illustrates the complexity of genetic epidemiology of deafness, and a possible interaction between *GJB2* 235delC and*⊿GJB6*-D13S1854 in nonsyndromic hearing impairment. Furthermore, 162 controls exhibited only wild-type alleles of *GJB6,* and none of the cases and controls screened showed *⊿GJB6*-D13S1830 mutation.

Mutations in several SLC family 26 genes are responsible for some distinct recessive disorders. *SLC26A4* (OMIM No. 600791) at 7q31 encodes a chloride-iodide transport protein expressed in the thyroid, kidney and inner ear. Its different mutations can lead to either syndromic deafness (Pendred syndrome) or non-syndromic recessive deafness. As shown in ***Table 4*** and ***Table 5***, *SLC26A4* mutation accounts for almost fifteen percent causative factors of hearing loss. In addition, mutations of *SLC26A4* usually cause enlarged vestibular aqueduct or cochlear deformity, which can be diagnosed with high-resolution CT scan of the temporal bone. In our study, deafness-associated mutations in *SLC26A4* were screened. There are 3 kinds of mutations in the *SLC26A4* gene (IVS7-2A>G, IVS7-2A>G+2168A>G, and 2167C>G) in the case group. A novel mutation, 2167C>G in exon 19, leading to a His–to-Asp substitution, was found.

Cochlear outer hair cells change their length in response to variations in membrane potential. This capability is believed to enable the sensitivity and frequency selectivity of the mammalian cochlea. Prestin is a transmembrane protein required for cochlear electromotility. This makes *SLC26A5* (OMIM No. 604943), the restricted expression of prestin in the outer hair cells of the cochlea, a strong candidate for human deafness[8]. Indeed, a single nucleotide change, IVS2-2A>G (NM_198999.1:c.-53-2A>G), in the second intron of *SLC26A5* has been reported in association with NSHL[9]. It was, however, observed only in the Caucasian probands with the carrier frequency of about 4.10%. The *SLC26A5* IVS2-2A>G sequence variation was not detected in Asians or African Americans according to previous reports. In this study, the IVS2-2A>G variant was either not found in a total of 297 Chinese Han people with either impaired or normal hearing by sequence analysis. The results indicated a special ethnic background between this sequence variation and human hearing loss.

Although the majority of cases with hereditary hearing loss are caused by nuclear gene defects, it has become clear that mtDNA (OMIM No. 561000) mutations can also cause deafness. Among the identified non-syndromic deafness-causing mtDNA mutations are A1555G, C1494T, T1095C, A827G and mutations at position 961 in the *12SrRNA*, and A7445G, 7472insC, T7510C, T7511C, T7512C and G7444A in the *tRNA^Ser(UCN)^*. Currently, it is estimated that these mutations are present in about 3.10% of patients with NSHL, but it is expected that this number will increase as genetic testing becomes more readily available[4],[9].

Similar to that of the *SLC26A5* IVS2-2A>G mutation, the association between mtDNA mutation and hearing loss has also a special ethnic difference. In the Asian nonsyndromic hearing-impaired populations, the incidence of the mtDNA mutation appears to be higher than in Caucasians as indicated by previous reports and as shown in ***Table 4*** and ***Table 5***. In our observation on 135 hearing-impaired subjects, the deafness-causing mtDNA mutations were detected in 8.14% (11/135) of the patients, a relatively high rate of incidence. Interestingly, these mitochondrial mutations were only found in the *12SrRNA*, and no deafness-associated mutations in *tRNA^Ser(UCN)^* were identified.

In summary, current data revealed that near half of the patients with NSHL carry deafness-causing mutation in *GJB2, GJB3, GJB6, SLC26A4*, or mtDNA *12SrRNA* genes: 43.57% in China (***Table 4***) and 50.06% in other countries (***Table 5***). Although the racial background is different, the results come to converge. As for causative factors, mutation in *GJB2* is the most common, followed by the *SLC26A4* variation. Other genes, such as *GJB3*, *GJB6* and mtDNA *12SrRNA*, may also play an important part in the pathogenesis of hearing loss in different countries or areas. These results indicate the necessity of genetic screening for mutations of these genes in patients with nonsyndromic deafness.
